# A chemical steering wheel for micromotors

**DOI:** 10.1093/nsr/nwab119

**Published:** 2021-07-06

**Authors:** Thomas E Mallouk

**Affiliations:** Department of Chemistry, University of Pennsylvania, USA

Molecular and colloidal ‘motors’ have attracted much attention over the past two decades as synthetic analogues of motor proteins and microorganisms such as bacteria. While schemes have been developed for powering these objects with light, ultrasound, electric and magnetic fields, and thermal gradients, chemically propelled swimmers most closely resemble their biological counterparts by converting chemical energy to mechanical energy [1]. The chemical gradients generated by one swimmer can be felt by its neighbors, and this gives rise to biomimetic collective behavior such as swarming and predator-prey interactions [2].

One of the most interesting of these behaviors is chemotaxis, which is the tendency to swim up or down a solute concentration gradient. Chemotaxis has been observed with a wide variety of chemically-powered swimmers, ranging from individual enzyme molecules to catalytic colloidal particles [3,4]. Catalytically driven chemotaxis has been implicated in the assembly of metabolons, which are intracellular clusters of enzymes [5]. It has also been studied for application in transporting drug molecules across the blood-brain barrier [6] and in separating active from inactive forms of enzyme molecules [7].

**Figure 1.**
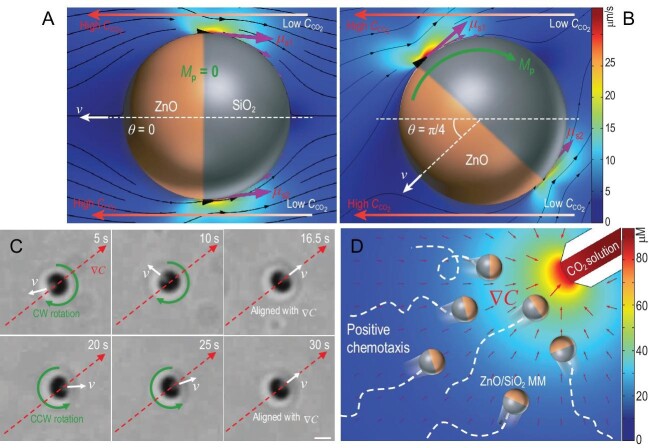
(A) A zinc oxide/silicon oxide Janus sphere is powered by a reaction with CO_2_, which generates Zn^2+^ and HCO_3_^–^ ions. (B) In a gradient of CO_2_ concentration, the reaction also generates a torque, M_p_, resulting in (C) particle re-alignment and (D) strong chemotaxis towards regions of high CO_2_ concentration [8].

Flagellar bacteria and other living microorganisms have sophisticated mechanisms for steering in gradients of signaling molecules, and this makes their chemotaxis towards nutrients or away from predators efficient. Synthetic swimmers can move just as fast, but they are constantly re-oriented by collisions with molecules in the fluid, i.e. by Brownian forces. Their chemotaxis can be understood as a random diffusional process that is biased by binding to reactant molecules, and is thus a weak effect [3]. Mou *et al.* [8] now show that strong chemotaxis can be achieved with synthetic micro-swimmers by designing them to compensate for Brownian rotation (Fig. 1). Effectively, the difference in reaction rates across the surface of a spherical swimmer can create a torque that continuously steers it into the gradient of fuel.

The fuel in this case is carbon dioxide, a non-toxic molecule that is the product of aerobic respiration. This suggests possible *in vitro* or even *in vivo* application, such as targeted drug delivery that would be sensitive to the metabolic differences between cells or tissues. Here though there is a potential problem, because the propulsion mechanism proposed by Mou *et al.* involves a chemically generated electric field. Electric fields are damped in ‘salty’ media such as biological fluids. Nevertheless, it may be possible to combine chemical steering with another bio-friendly propulsion mechanism, e.g. by using ultrasound as the power source. This strategy has been used to propel micro-swimmers up- or downstream in flows, with the chemical reaction acting like the tail of a kite to orient rod-shaped particles [9]. Given the variety of propulsion mechanisms now available to micro-swimmers, there is significant potential to develop new applications based on chemical steering.

***Conflict of interest statement*.** None declared.
